# Mapping a protein recognition centre with chiral photoactive ligands. An integrated approach combining photophysics, reactivity, proteomics and molecular dynamics simulation studies†
†Electronic supplementary information (ESI) available: Experimental section, additional computational calculations, figures and references. See DOI: 10.1039/c6sc04900a



**DOI:** 10.1039/c6sc04900a

**Published:** 2017-01-05

**Authors:** Daniel Limones-Herrero, Raúl Pérez-Ruiz, Emilio Lence, Concepción González-Bello, Miguel A. Miranda, M. Consuelo Jiménez

**Affiliations:** a Departamento de Química/Instituto de Tecnología Química UPV-CSIC , Universitat Politècnica de València , Camino de Vera s/n , 46022 , Valencia , Spain . Email: mmiranda@qim.upv.es ; Email: mcjimene@qim.upv.es; b Centro Singular de Investigación en Química Biolóxica e Materiais Moleculares (CIQUS) , Departamento de Química Orgánica , Universidade de Santiago de Compostela , C/ Jenaro de la Fuente s/n , 15782 Santiago de Compostela , Spain

## Abstract

A multidisciplinary strategy to obtain structural information on the intraprotein region is described here. As probe ligands, (*S*)- and (*R*)-**CPFMe** (the methyl esters of the chiral drug carprofen) have been selected, while bovine α_1_-acid glycoprotein (BAAG) has been chosen as a biological host. The procedure involves the separate irradiation of the BAAG/(*S*)-**CPFMe** and BAAG/(*R*)-**CPFMe** complexes, coupled with fluorescence spectroscopy, laser flash photolysis, proteomic analysis, docking and molecular dynamics simulations. Thus, irradiation of the BAAG/**CPFMe** complexes at *λ* = 320 nm was followed by fluorescence spectroscopy. The intensity of the emission band obtained after irradiation indicated photodehalogenation, whereas its structureless shape suggested covalent binding of the resulting radical **CBZMe˙** to the biopolymer. After gel filtration chromatography, the spectra still displayed emission, in agreement with covalent attachment of **CBZMe˙** to BAAG. Stereodifferentiation was observed in this process. After trypsin digestion and ESI-MS/MS, the incorporation of **CBZMe** was detected at Phe68. Docking and molecular dynamics simulation studies, which were carried out using a homology model of BAAG, reveal that the closer proximity of the aromatic moiety of the (*S*)-enantiomer to the phenyl group of Phe68 would be responsible for the experimentally observed, more effective chemical modification of the protein. The proposed tridimensional structure of BAAG covalently modified by the two enantiomers is also provided. In principle, this approach can be extended to a variety of protein/ligand complexes.

## Abstract 2

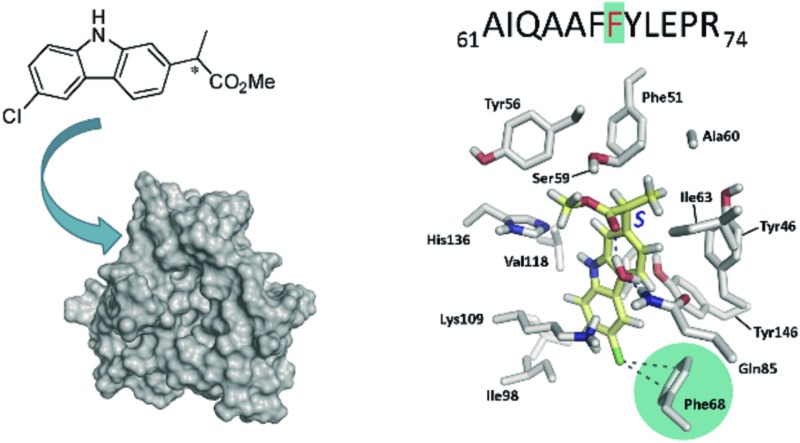
Photobinding of **CPFMe** to Phe68 reveals the structural details of the recognition centre of BAAG for this ligand.

## Introduction

The intraprotein regions of proteins are not passive receptacles for substrate binding, but very complex microenvironments that employ a wide variety of physicochemical interactions (van der Waals forces, hydrogen bonding, *etc.*) for the complexation of substrates. The sites are formed by side chains of specific amino acid residues in a particular three-dimensional arrangement that play an important role in the accommodation of ligands. Besides the favourable structural requirements, stabilising ligand–protein interactions are necessary to form complexes. These interactions form the basis of molecular recognition, which is essential for most of the biological functions of these biomacromolecules, such as biodistribution (transport proteins), biotransformation (enzymes) or activation/deactivation (receptors).^1^

Experimentally, precise structural information can be obtained from techniques such as cryo-EM or X-ray crystallography, when suitable crystals of the ligand–protein complex are obtained with the appropriate diffracting properties.^2^ However, the development of alternative and structurally informative methodologies is still highly desirable. In principle, a wide variety of ligands contain photoreactive chromophores whose excitation could give rise to very short-lived excited states or reactive intermediates and are capable of undergoing covalent photobinding to amino acid residues.^3^ As these species have negligible capability of diffusing away from the place where they are generated, photoreaction has to take place in close proximity to them. Thus, identification of the molecular recognition centre would be achieved by photoinduced covalent modification of key amino acid residues by the ligand. This is reminiscent of photoaffinity labelling, but without the need to carry out time consuming chemical syntheses of reactive derivatives, which in addition may present binding affinities different to those of the unaltered ligands.^4^

Among the ligands containing photoreactive chromophores, a number of xenobiotics (drugs, cosmetics, *etc.*) have been reported, which possess the ability to induce changes in the sensitivity of a subject with respect to UV-Vis radiation, whether of solar or artificial origin. Such a phenomenon, called photosensitivity, occurs when the photoexcited xenobiotic is able to interact with cellular components of an organism, especially in the skin.

Drug-induced photosensitivity includes photoallergic reactions, which are thought to involve covalent drug–protein photobinding, leading to the formation of a complete antigen that may trigger a hypersensitivity reaction due to a cell-mediated immune response.^5^ Hence, transport proteins have been employed as models to investigate the photobinding process. Most of the work that has been done uses serum albumins,^6^ but very little is known on other relevant transport proteins, such as α_1_-acid glycoproteins (AAGs). In general, the existing studies are limited to detecting the irreversible incorporation of the drug chromophore to the protein by means of gel filtration chromatography followed by absorption or emission spectroscopy, but they do not provide structural information.^7^

The goal of the present work is to obtain structural information on the recognition centre of AAGs by exploiting the photobinding of a ligand. Several activities of physiological significance have been described for AAGs, such as their ability to bind and transport small hydrophobic molecules and to act as immuno-modulating agents. The AAGs’ serum concentrations remain constant under normal physiological conditions but increase markedly during acute-phase reactions, in response to systemic tissue injury, inflammation or infection. Thus, AAGs are considered as major members of the positive acute phase protein family. Specifically, bovine α_1_-acid glycoprotein (BAAG) has been chosen as the biological host due to its ability to bind and transport endogenous or exogenous ligands, with basic or neutral character, mainly in one large and flexible binding site. The protein consists of one polypeptide chain and is highly glycosylated; the BAAG molecular weight has been determined as 33.8 kDa by MALDI-TOF mass spectrometry analysis.^8^

**Fig. 1:**
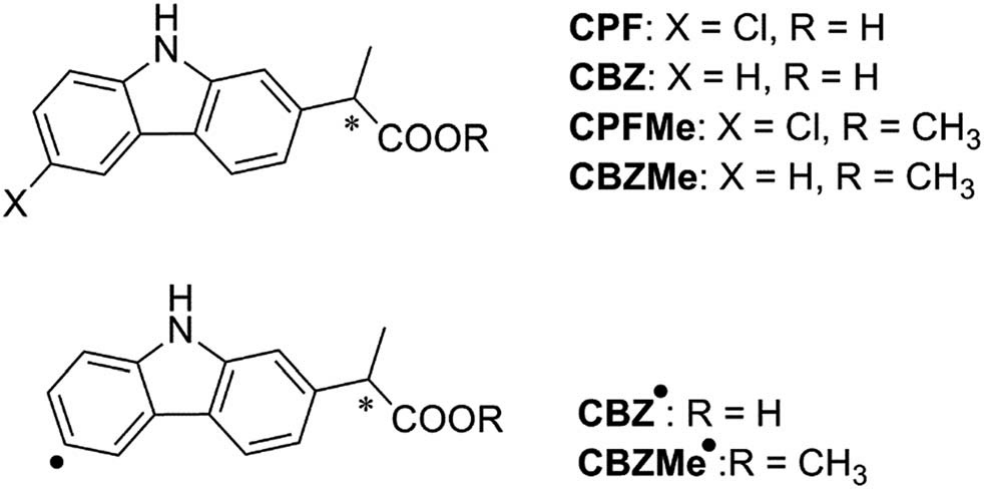
Chemical structures of **CPF** and derivatives.

With regard to the probe ligand, **CPFMe**, the two enantiomers of the methyl ester of the chiral non-steroidal anti-inflammatory drug carprofen (**CPF**) have been selected (see chemical structures in Fig. 1). Introduction of the methyl group in arylpropionic acids, such as in **CPFMe**, leads to substrates that should bind to BAAG more efficiently than the parent drug.^9^ It has been reported that **CPF** is able to induce photoallergy, manifested as photocontact dermatitis.^10^ The effects were initially observed in patients under medical treatment and then, after introduction for veterinary purposes, in factory workers producing the drug and in pet owners. The main photoreaction of **CPF** in aqueous solution is dehalogenation, a process that generates highly reactive aryl radicals (**CBZ˙**, Fig. 1) capable of causing irreversible covalent chemical modification of proteins. Alternatively, in the presence of the appropriate hydrogen donors, the product resulting from dehalogenation is **CBZ** (Fig. 1).^11^

Specifically, the strategy employed in this work involves the separate irradiation of the two enantiomers of **CPFMe** encapsulated within BAAG, to obtain their respective covalent adducts, coupled with a multidisciplinary methodology that comprises fluorescence and transient absorption spectroscopies, proteomic analysis and docking and molecular dynamics simulation studies. The results clearly show that after the dehalogenation of **CPFMe**, in order to afford **CBZMe˙**, irreversible covalent modification of BAAG occurs to Phe68. Moreover, the process is stereoselective, with higher chemical modification of the protein for the (*S*)-enantiomer.

## Results and discussion

The required (*S*)- and (*R*)-enantiomers of **CPFMe** were prepared from their corresponding enantiopure free acids, (*R*)- and (*S*)-**CPF**, which were separated from the commercial racemic mixture by chiral HPLC, and then subjected to esterification with methanol to afford (*R*)- and (*S*)-**CPFMe** (see details in the ESI†).

### Photophysical studies

Since the photophysical properties of a given chromophore may be strongly dependent on the experienced microenvironment, a thorough photophysical study including fluorescence spectroscopy and laser flash photolysis was performed on the BAAG/**CPFMe** complexes.

**Fig. 2:**
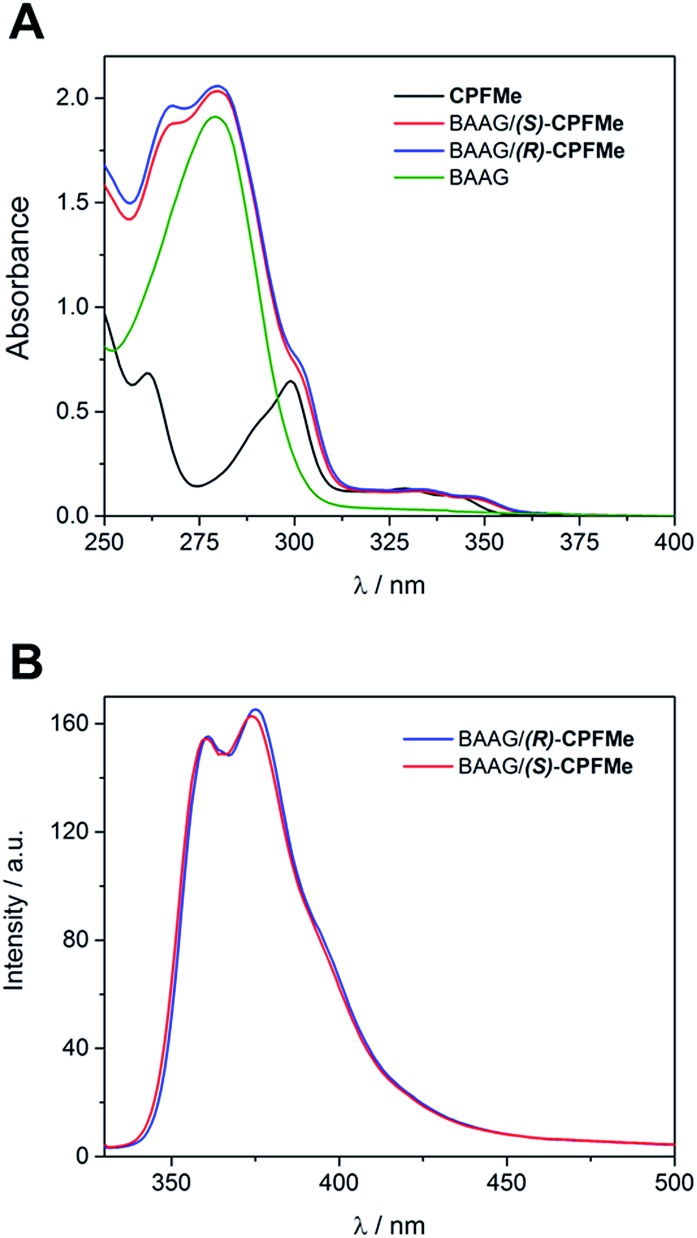
(A) Absorption spectra of **CPFMe** (in MeCN), BAAG, BAAG/(*R*)-**CPFMe** and BAAG/(*S*)-**CPFMe** in PBS. (B) Fluorescence spectra of BAAG/(*R*)-**CPFMe** and BAAG/(*S*)-**CPFMe** in PBS/air at *λ*_exc_ = 320 nm.

Solutions of (*S*)- and (*R*)-**CPFMe** (3.3 × 10^–5^ M) were prepared in the presence of 2 equivalents of BAAG, in phosphate-buffered saline (PBS). Fig. 2A shows the absorption spectra of BAAG/(*S*)-**CPFMe** and BAAG/(*R*)-**CPFMe**; the maxima in the UVB-UVA region appear at 300, 334 and 348 nm. The spectra of the isolated **CPFMe** (in MeCN, due to its poor solubility in PBS) and BAAG are shown as controls. The fluorescence spectra of the BAAG/**CPFMe** complexes at *λ*_exc_ = 320 nm consist of a band with two maxima at 360 and 375 nm (Fig. 2B).

**Fig. 3:**
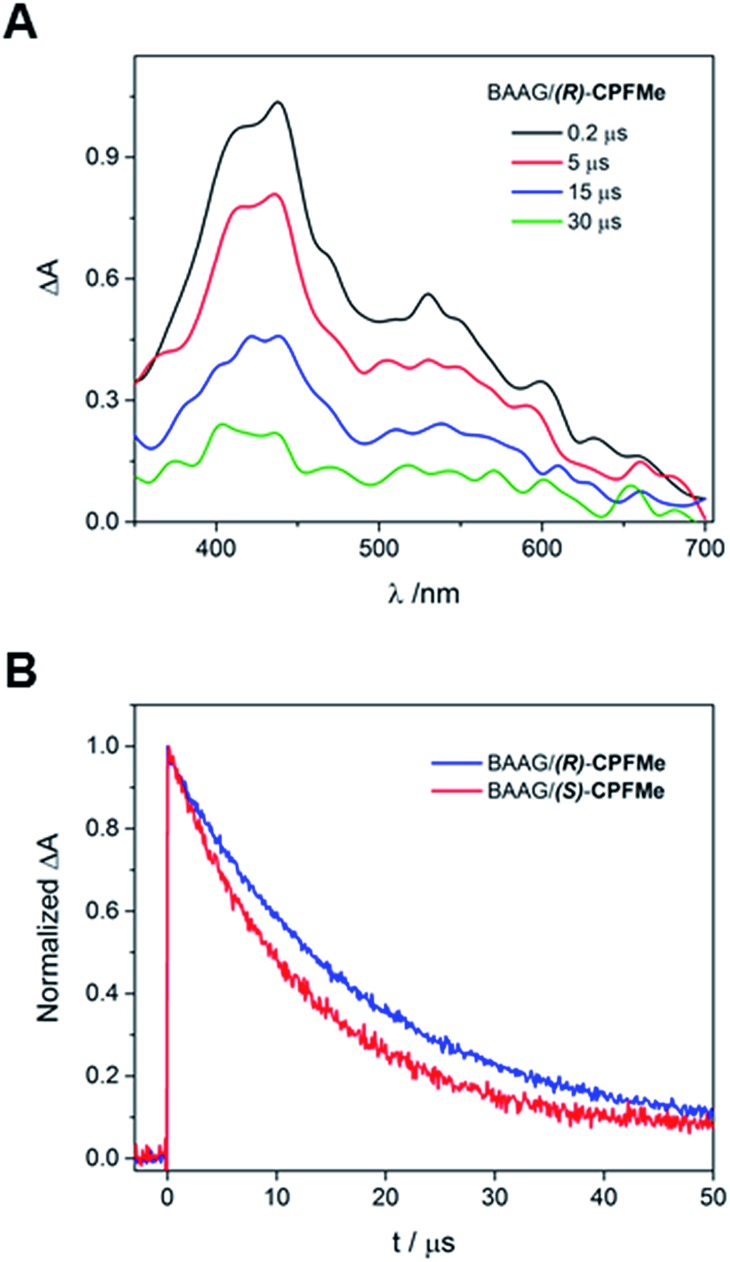
Laser flash photolysis of BAAG/**CPFMe** at *λ*_exc_ = 355 nm, in PBS/air. (A) Transient absorption spectra in different time windows for (*R*)-**CPFMe**. (B) Normalised decay kinetic traces at *λ*_mon_ = 450 nm for both enantiomers. [**CPFMe**] = 3.3 × 10^–4^ M, [BAAG] = 6.6 × 10^–4^ M.

Laser flash photolysis of BAAG/(*S*)-**CPFMe** and BAAG/(*R*)-**CPFMe** was performed at *λ*_exc_ = 355 nm. The results in different time windows for the (*R*)-enantiomer are shown in Fig. 3A. Thus, a broad band with maximum at 450 nm was observed, attributed to the triplet excited state (^3^ CPFMe*), confirmed by the literature data for **CPF** in MeCN.^7*c*^

For the (*S*)-enantiomer, the shapes of the spectra were similar. However, stereodifferentiation was observed in the decay kinetics of BAAG/(*R*)-**CPFMe** and BAAG/(*S*)-**CPFMe** monitored at *λ* = 450 nm (Fig. 3B). Thus the triplet lifetime (*τ*_T_) value for the (*S*)-enantiomer was much shorter (13 μs) than that of the (*R*)-analog (18 μs). This could be related to the more efficient quenching of ^3^ CPFMe* by electron transfer from Trp in the case of (*S*)-**CPFMe**, which is in agreement with a closer distance from (*S*)-**CPFMe** to a Trp residue than in the case of (*R*)-**CPFMe**, within the binding site pocket.^12^ Stereodifferentiation in the triplet lifetimes of ligands within transport proteins has previously been observed in other cases, such as carprofen, flurbiprofen, flurbiprofen methyl ester or 2-anthracene propionic acid incorporated into serum albumins.^7c,14^

**Fig. 4:**
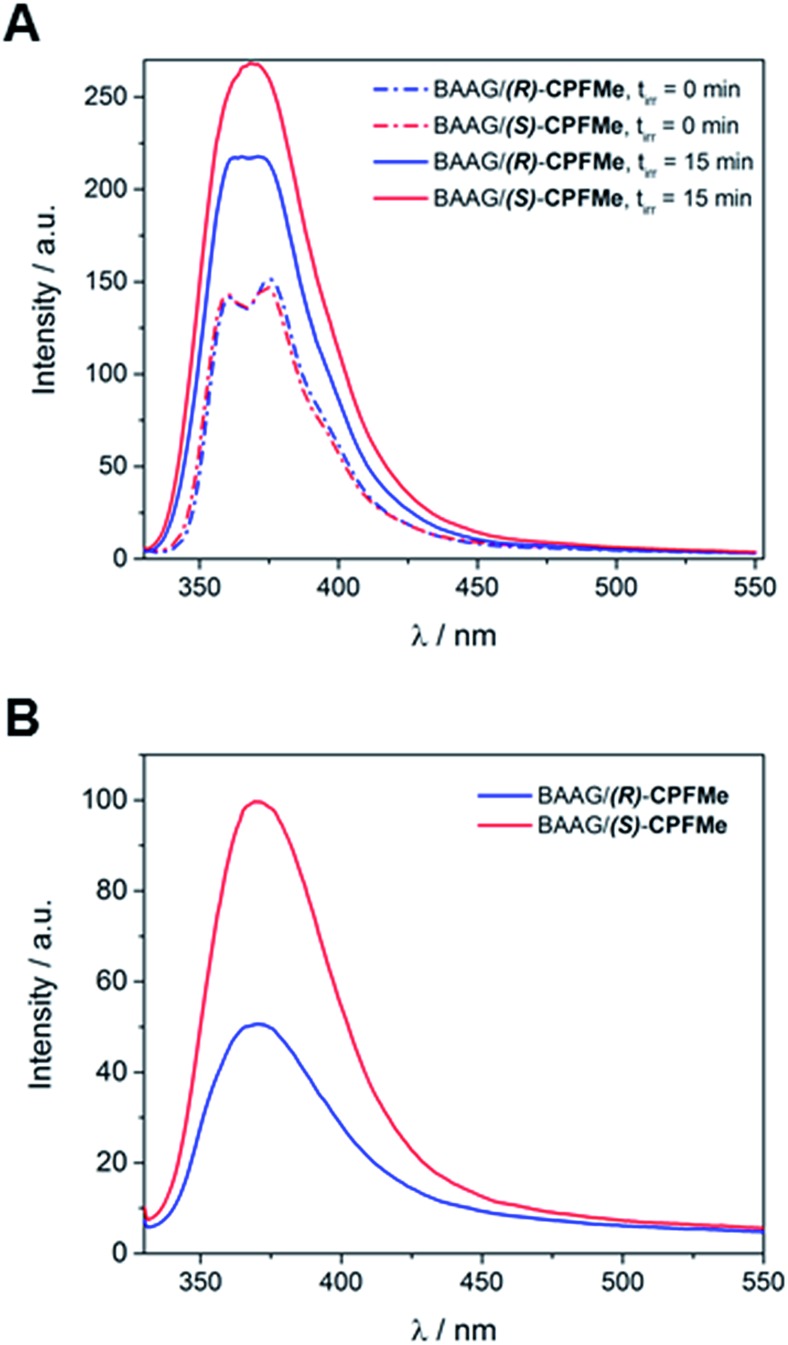
(A) Fluorescence spectra of non- and 15 min-irradiated samples of BAAG/**CPFMe**. (B) Fluorescence spectra of 15 min-irradiated samples of BAAG/**CPFMe** after GndCl treatment and filtration through Sephadex. [**CPFMe**] = 3.3 × 10^–5^ M, *λ*_exc_ = 320 nm, [BAAG] = 6.6 × 10^–5^ M. Experiment errors were lower than 5% of the obtained values.

Solutions of BAAG/(*S*)-**CPFMe** and BAAG/(*R*)-**CPFMe**, again at a 2 : 1 protein/**CPFMe** molar ratio, were irradiated separately at *λ* = 320 nm, and the process was followed by fluorescence spectroscopy. The spectra recorded before and after 15 minutes of irradiation are shown in Fig. 4A. For both **CPFMe** enantiomers, the emission bands of the irradiated samples did not exhibit the characteristic fine structures with two well-defined maxima of **CPFMe**, and were both more intense. This is in agreement with the occurrence of the photodehalogenation reaction leading to products with fluorescence quantum yields much higher than that of **CPFMe**.^13^

To check whether covalent modification of the protein was occurring, the photolysates were treated with 6 M of guanidinium chloride and filtered through Sephadex (details in ESI†), a process that allows the removal of the species with the lower molecular weight. The obtained spectra are shown in Fig. 4B. Clearly, emission is still observed, indicating that this **CBZMe**-derived species is covalently attached to BAAG. In parallel, non-irradiated samples of BAAG/**CPFMe** were subjected to the same work up as control experiments. As expected for the non-protein bound complexes, no emission was observed in these cases.

Interestingly, the **CPFMe** binding to BAAG resulted in being stereoselective, which would be in agreement with the relative lifetime values obtained by LFP.

### Proteomic analysis

**Fig. 5:**
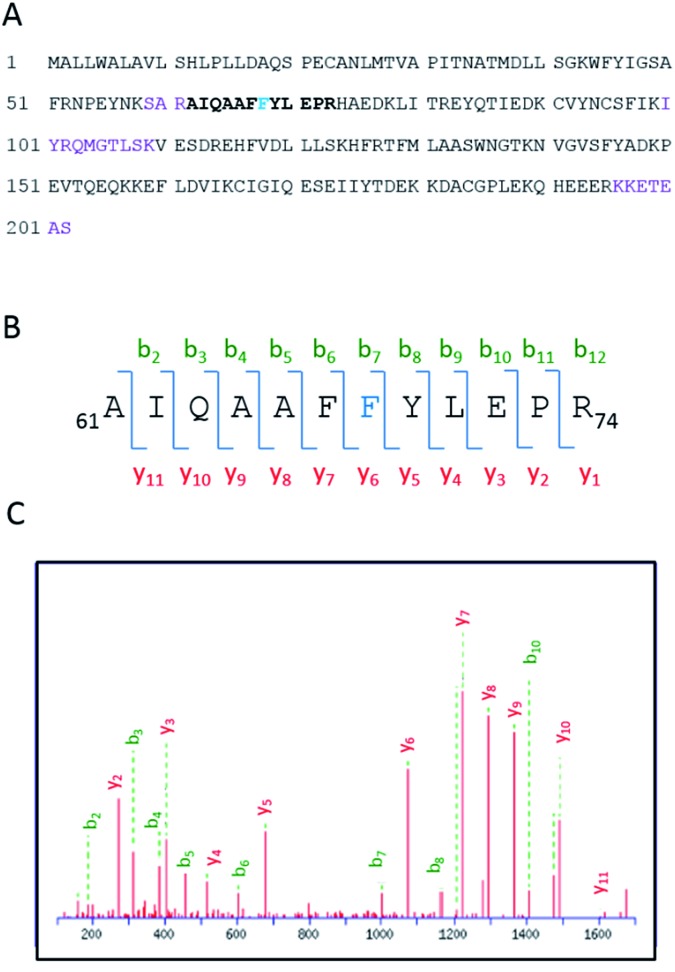
(A) Amino acid sequence of BAAG. The modified peptide is emboldened, and the altered amino acid residue is indicated in blue. (B) Modified peptide and fragmentation keys. (C) ESI-MS/MS spectra and fragmentation pathway of the **CBZMe**-modified peptide.

Having demonstrated the photobinding of **CPFMe** to BAAG by fluorescence measurements, the formation of covalent photoadducts was investigated in more detail by proteomic analysis. These studies would provide precise information of which amino acid(s) were covalently modified. Thus, the photoreactivity of the two enantiomers of **CPFMe** with BAAG was addressed by HPLC-nanoESI analysis. The irradiated BAAG/(*S*)-**CPFMe** and BAAG/(*R*)-**CPFMe** mixtures were filtered to remove excess ligand; then underwent trypsin digestion, which cleaves the peptide chain mainly at the carboxyl side of Lys or Arg residues (unless they neighbour a Pro residue), and finally HPLC-MS/MS analysis was performed. This was intended to obtain information on the modified peptide sequence and to characterize the adduct. Full scan and fragmentation data files were analysed using the Mascot® database search engine (Matrix Science, Boston, MA, USA) and by entering variable modifications that take into account the main possible residues (FWY) able to react with the carbazolyl radical **CBZMe˙** obtained after dehalogenation of **CPFMe**. The sequence coverage was 90% for BAAG/(*R*)-**CPFMe** and 93% for BAAG/(*S*)-**CPFMe**. The results for the (*R*)-enantiomer are shown in Fig. 5A, with the non-matched peptides indicated in violet.

The main result confirmed the identification of only one **CBZMe**–BAAG derived peptide adduct, _61_AIQAAFFYLEPR_74_, for both **CPFMe** enantiomers (Fig. 5B).

The modification site of the peptide was assessed by tandem mass experiments on the trypsin digests. The ESI-MS/MS spectra and fragmentation pattern are shown in Fig. 5C. Their related data are summarised in Table 1. The peptide sequence agreed well with the “y” and “b” ion series. The MS/MS fragment ions showed an unmodified y ion series from y_1_ to y_5_, whereas an increment of *m*/*z* 398 was detected at Phe68 between y_5_ and y_6_ corresponding to **CBZMe˙**–Phe˙(–H_2_O). Accordingly, the b ion series suffered the same increase from b_6_ to b_7_. Thus, the modified amino acid is Phe68.

**Table 1:** Identification of the **CBZMe**-modified peptide by MS/MS spectrometry

Amino acid	y	*m*/*z*	b	*m*/*z*
A			b_1_	72.044
I	y_11_	1605.8100	b_2_	185.1285
Q	y_10_	1492.7260	b_3_	313.1870
A	y_9_	1364.6674	b_4_	384.2241
A	y_8_	1293.6303	b_5_	455.2263
F	y_7_	1222.5932	b_6_	602.3297
F	y_6_	1075.5247	b_7_	1000.4927
Y	y_5_	677.3617	b_8_	1163.5560
L	y_4_	514.2984	b_9_	1276.6401
E	y_3_	401.2143	b_10_	1405.6827
P	y_2_	272.1717	b_11_	1502.7355
R	y_1_	175.1190		

**Scheme 1:**
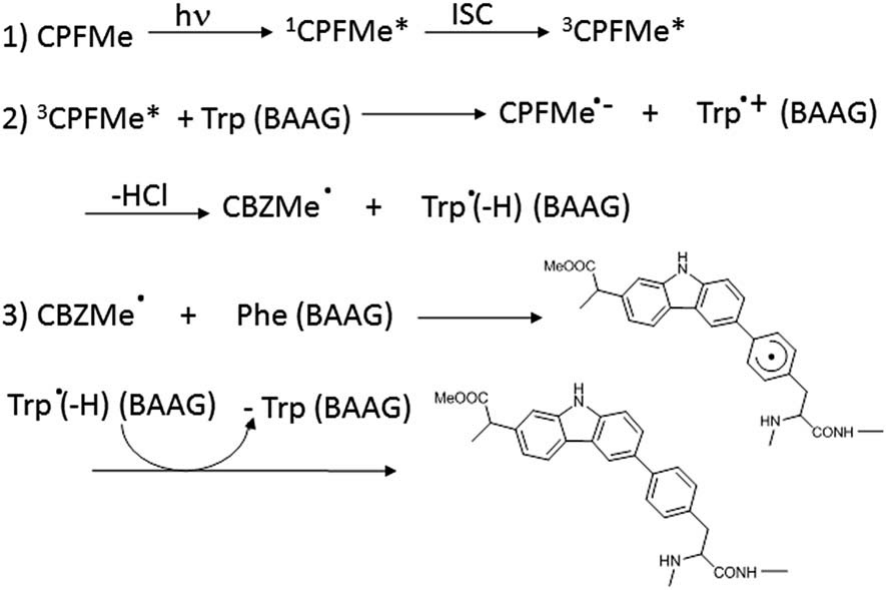


In order to ascertain whether Phe can react directly with ^3^ CPFMe*, a triplet quenching experiment was performed. Thus, LFP of **CPFMe** in acetonitrile at *λ*_exc_ = 355 nm was run in the presence and also in the absence of Phe methyl ester, but no differences were found in the triplet lifetimes. In contrast, it has been previously established that quenching by Trp occurs at diffusion controlled rates.^12^ Combining this information with the proteomic results, a plausible mechanism is proposed (Scheme 1). It involves the reaction of ^3^ CPFMe* with Trp to afford the corresponding radical ion pairs; the **CBZMe˙** radical formed after the loss of Cl^–^would react with Phe, resulting in covalent binding to this residue.

### Molecular modelling

Due to (i) the specific covalent modification of Phe68 in BAAG by (*S*)- and (*R*)-**CPFMe** and (ii) reasoning that the differences observed between the two enantiomers would be due to the binding mode of these ligands in the recognition centre of the protein, computational studies were performed. To this end, molecular docking using GOLD 5.2 (ref. 15) was carried out using a homology model of BAAG. As no three-dimensional structure of BAAG is available, a homology model was constructed using the Phyre2 homology modelling web server^16^ (see a detailed description in the ESI, pp. S9 and S10†). A comparison of the crystal structure of the HAAG/(*R*)-1-glycerol acetate complex (PDB code ; 3KQ0 (ref. 17)) and the constructed three-dimensional structure of apo-BAAG reveals that its recognition centre is a large apolar pocket (Fig. S1A and B†). It mainly involves Leu70, Ile98, Leu107, Val118, Leu131, Phe68, Phe129, and Trp44. As for the HAAG protein, the pocket is closed by an electrostatic interaction between two polar residues, which for BAAG are Lys109 and Glu83. In addition, the recognition centre includes another aromatic residue, specifically, Tyr146. More importantly, the experimentally observed covalently modified Phe68 residue is located in close contact with Trp44, which would trigger the photocleavage reaction. A second phenylalanine residue, Phe129, is also located in the vicinity of Trp44.

With the BAAG homology model in hand, docking studies were then performed. The proposed binding modes were further analysed by Molecular Dynamics (MD) simulation studies in order to assess the stability and therefore the reliability of the postulated binding. The results of these studies are discussed below.

**Fig. 6:**
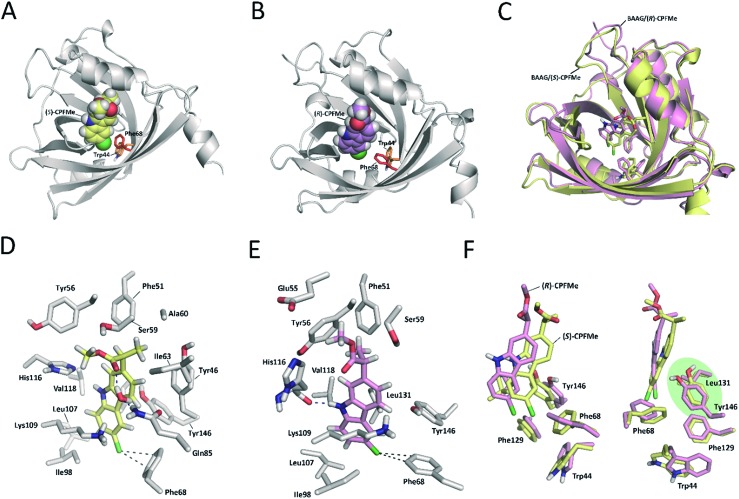
Binding modes of (*S*)- (A and D) and (*R*)-**CPFMe** (B and E) obtained by docking and MD simulation studies of the recognition centre of the BAAG protein (grey). The viewpoints showed for the ligands (*S*)-**CPFMe** (yellow) and (*R*)-**CPFMe** (pink) correspond to snapshots after 50 ns of MD simulation. (C and F) Comparisons of the binding modes of (*S*)- (yellow) and (*R*)-**CPFMe** (pink) in the recognition centre of the BAAG protein. Relevant side chain residues are shown and labelled. Polar (blue) contacts and distances (grey) to Phe68 are shown as dashed lines. Note how the C6 atom of the two isomers is located close to Phe68 and how Tyr146 and Leu131 side chains prevent the covalent modification of Phe129.

Docking and MD simulation studies were carried out using GOLD 5.2 (ref. 15), with the protein geometries found in the apo-BAAG homology model. The position of (*R*)-1-glycerol acetate in PDB code ; 3KQ0 was used to define the active-site and the radius was set to 8 Å. The proposed binding modes of (*S*)- and (*R*)-**CPFMe** were further analysed by MD simulation studies. As expected, at the beginning of the simulation (the first ∼25 ns) a small displacement of the two ligands was observed resulting from the initial adjustment of the bulky ligands with flexible side chains (ester) into apo-structures (Fig. S2†). However, eventually the complexes revealed to be stable during most of the simulation (∼75 ns) with the aromatic moiety located in close proximity to Phe68 (Fig. 6).

**Fig. 7:**
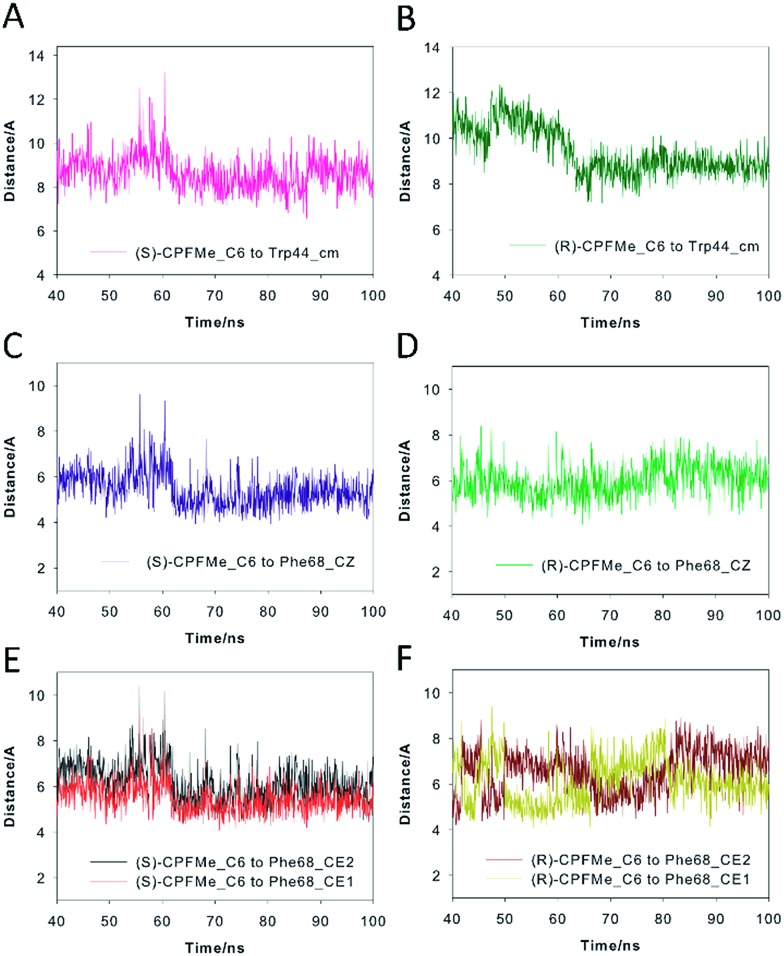
Variation of the relative distances between the C6 atom of (*S*)-**CPFMe** (A, C and E) and (*R*)-**CPFMe** (B, D and F) and the CZ atom (*para*-), the CE1 and CE2 atoms (*meta*-) of Phe68 and the centre of mass of Trp44 in the corresponding BAAG/**CPFMe** protein complexes during the last 60 ns of the simulation. Note how the C6 atom of (*S*)-**CPFMe** remains closer to Phe68 than its enantiomer during the simulation.

Our computational studies revealed that the binding mode of the two enantiomers would be in general quite similar in terms of the type of amino acids involved and the kind of interactions with them. The binding free energies of each ligand were calculated using the MM/PBSA^18^ approach in implicit water (generalised Born, GB) as implemented in Amber. (*S*)-**CPFMe** has a binding free energy that is –0.9 kcal lower than that of (*R*)-**CPFMe** (Table S1†). Similar results were obtained using the water-swap application.^19^ In particular, the aromatic moiety of the ligands would establish lipophilic interactions with the side chains of residues Ile98, Leu107, Val118, Ile63, and Lys109 (carbon chain) and CH–π interactions with the side chain of Leu114 (Fig. 6D and E). Besides this similarity involving the aromatic ring of the ligands, the stereochemistry of the flexible side chain proved to be a key element in the proximity of the C6 carbon atom of the two enantiomers to the phenyl group of the experimentally observed covalently modified Phe68, which would explain its different behaviour (Fig. 6C and F). Whereas for (*S*)-**CPFMe**, the position of the ester chain is anchored in the recognition centre by hydrogen bonding with the side chain of Gln85 *via* a water molecule, however, for (*R*)-**CPFMe** the chain is quite flexible. Gln85 is located in the vicinity of Phe68. In addition, the NH group of the aromatic moiety in (*R*)-**CPFMe** would interact by a strong hydrogen bonding interaction with the main carbonyl group of His116 and the methyl group of the side chain would be located in the apolar pocket, involving Phe34 and Tyr39. These residues are located in the opposite site of Phe68. As a consequence of these attractive interactions, (*R*)-**CPFMe** and therefore its C6 carbon atom would be located further from the phenyl group of Phe68 than in the case of (*S*)-**CPFMe**. This displacement of (*R*)-**CPFMe** towards the opposite site of the recognition centre would explain the less efficient covalent modification of the Phe68 residue (Fig. 6F). Analysis of the contribution of each residue to the binding free energy carried out with the water-swap^19^ application also revealed the interaction of the (*S*)-enantiomer with Phe68 would be 8-fold stronger than its enantiomer (Fig. S3†). A study of the relative distances between the C6 atom in the two ligands and the CZ (*para*) and CE1 and CE2 (*meta*) carbon atoms in Phe68 during the simulation is shown in Fig. 7. For (*S*)-**CPFMe** in the last 60 ns of simulation, the average distances between the C6 atom and the CZ, CE1 and CE2 carbon atoms in Phe68 were found to be 5.4, 5.4 and 6.0 Å, respectively (Fig. 7C and E). For (*R*)-**CPFMe**, the average distances were 6.0, 6.0 and 6.6 Å, respectively (Fig. 7D and F). It is important to highlight that once the C–Cl bond cleavage occurs, the generated radical would be located closer to the phenyl ring of Phe68 as this would reduce steric hindrance.

The results of our computational studies also explain why the covalent modification of Phe68 is only observed in spite of the presence of another phenylalanine in the vicinity of the ligands, specifically, Phe129. The proximity and higher accessibility of Phe68 to the ligands would explain this fact. Thus, an analysis of the relative distance between the C6 carbon atom in the two ligands and the CZ (*para*) and CE1 and CE2 (*meta*) carbon atoms of Phe68 and Phe129 revealed that the ligands are closer to Phe68 than Phe129 (Fig. S4†). In addition, as shown in Fig. 7F, the proximity of Tyr146 and Leu131 to the aromatic rings of the two ligands would prevent reaction with Phe129, which is located behind those residues. Moreover, as expected, the covalently modified Phe68 is located in the vicinity of a tryptophan residue, specifically, Trp44 (Fig. 7B). Our simulation studies showed that this residue is also located closer to the C6 carbon atom of (*S*)-**CPFMe** than its enantiomer during the simulation with an average distance of 8.6 Å *vs.* 9.2 Å for (*R*)-**CPFMe** during the last 60 ns of the simulation (Fig. 7A and B). The centre of mass of Trp44 was considered for these calculations.

**Fig. 8:**
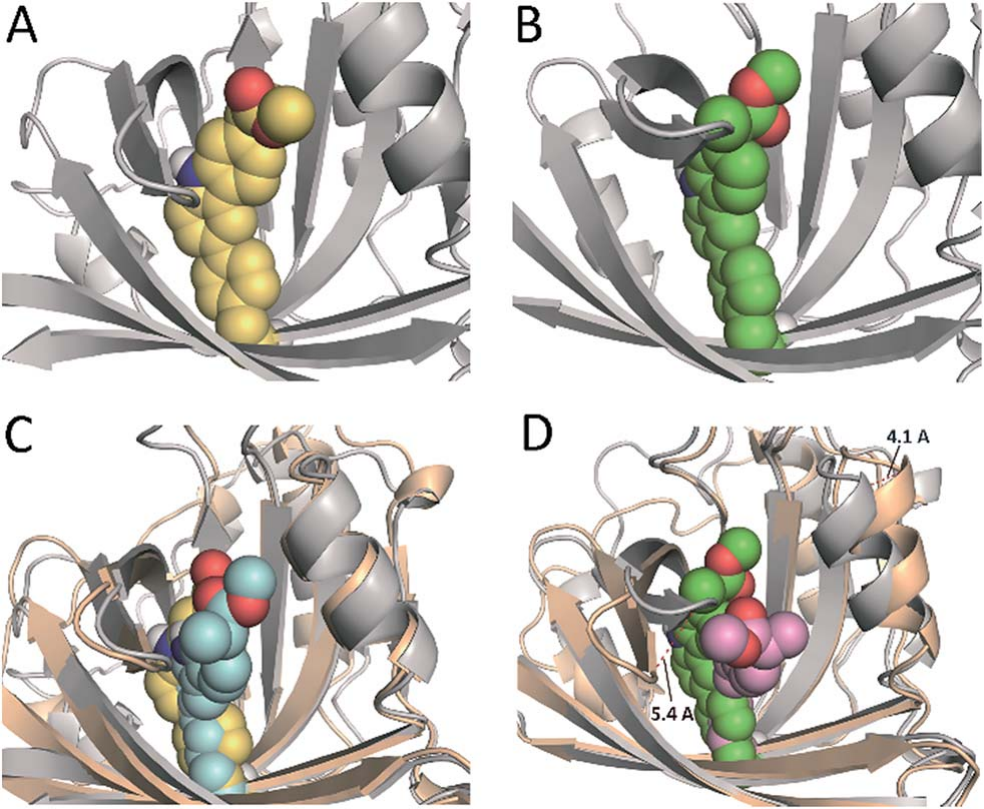
(A and B) Proposed tridimensional structure of BAAG covalently modified by (*S*)-**CPFMe** (A, yellow) and (*R*)-**CPFMe** (B, green) at the *para*-position of Phe68, obtained by MD simulation studies. (C and D) Comparison of the resulting *para*- [protein (grey)] and *meta*- [protein (wheat)] BAAG/(*S*)-**CPFMe** (C) and BAAG/(*R*)-**CPFMe** (D) adducts. *meta*-Covalently modified Phe68 residues by (*S*)- and (*R*)-**CPFMe** are shown as blue and pink spheres, respectively. The viewpoints shown correspond to snapshots after 70 ns of MD simulation. Note how for (*R*)-**CPFMe**, the *meta*-adduct causes a large opening of the recognition centre of up to 5.4 Å.

In an effort to obtain further details of the covalent modification of Phe68 by the two enantiomers, the possible insertion at its *para* and *meta* positions were then explored by MD simulation studies (Fig. 8 and S5†). For (*S*)-**CPFMe**, the analysis of the relative distances between the C6 carbon atom of the ligand and the CZ, CE1 and CE2 carbon atoms in Phe68 revealed that the two positions are equally accessible (Fig. 7C and E). In addition, no significant binding differences were observed in the two possible adducts (Fig. 8C). The two would be well embedded in the recognition centre. These findings suggest that, from a computational point of view, both *para* and *meta* adducts would be possible. On the contrary, the most plausible covalent modification of the Phe68 residue by the *R* enantiomer would be at its *para* position (Fig. 8B). In this case: (i) the CZ carbon atom of Phe68 would be located closer to the C6 carbon atom of the ligand than the CE1 and CE2 atoms (Fig. 7D *vs.* F); and (ii) the corresponding *meta* adduct would open the recognition centre up to 5.4 Å to accommodate the ester side chain in this arrangement (Fig. 8D).

## Conclusions

A multidisciplinary strategy has been employed here to obtain structural information on the intraprotein region. The principle has been proven by separate irradiation of BAAG/(*S*)-**CPFMe** and BAAG/(*R*)-**CPFMe** complexes, coupled with photophysical studies, proteomic analysis, and docking and molecular dynamics simulations. Overall, the obtained results indicate that the ligand is located in a large apolar pocket that mainly involves Leu70, Ile98, Leu107, Val118, Leu131, Phe68, Phe129, and Trp44. Steady-state irradiation of the complexes leads to the dehalogenation of **CPFMe**, ultimately resulting in the covalent photobinding of the intermediate aryl radical **CBZMe˙** to Phe68. This process, which is more efficient for the (*S*)-enantiomer, could be triggered by the neighbouring Trp44. Accordingly, the triplet excited state lifetime of the BAAG/(*S*)-**CPFMe** complex is shorter than that of BAAG/(*R*)-**CPFMe**. Thus, this strategy allows the identification of the molecular recognition centre of BAAG through the photoinduced modification of key amino acid residues by **CPFMe** and could, in principle, be extended to a variety of protein/ligand complexes. We believe that the studies reported here would be helpful for studying the recognition centre of diverse proteins with photoactive ligands. The only requirement for the methodology to be applicable is the presence of an active chromophore in the ligand that, after light absorption, can generate reactive species capable of attacking one or several amino acid residues, leading to the formation of covalent, irreversible adducts.
