# Supplementary material for: Mapping a protein recognition centre with chiral photoactive ligands. An integrated approach combining photophysics, reactivity, proteomics and molecular dynamics simulation studies
Source: Chem Sci. 2017 Jan 5;8(4):2621–8. doi: 10.1039/c6sc04900a (PMC5431658; doi:10.1039/c6sc04900a)
Supplement: Supplementary file 1 [file SC-008-C6SC04900A-s001.pdf]

Electronic Supplementary Material (ESI) for Chemical Science.
This journal is © The Royal Society of Chemistry 2017

Supporting information for

Mapping a protein recognition centre with chiral photoactive ligands. An integrated approach combining photophysics, reactivity, proteomics and molecular dynamics simulation studies

Daniel Limones-Herrero, Raúl Pérez-Ruiz, Emilio Lence, Concepción González-Bello, Miguel A. Miranda* and M. Consuelo Jiménez*

Table of Contents

1. **Figure S1**.......S2
2. **Figure S2**.......S3
3. **Table S1**.......S4
4. **Figure S3**.......S4
5. **Figure S4**.......S5
6. **Figure S5**.......S6
7. **Experimental Section**.......S7
   7.1 General.......S7
   7.2 Synthesis of (*R*)-CPFMe and (*S*)-CPFMe.......S7
   7.3 Fluorescence experiments.......S7
   7.4 Laser flash photolysis experiments.......S7
   7.5 Steady-state photolysis experiments.......S8
   7.6 Treatment with guanidinium chloride and filtration through Sephadex.......S8
   7.7 Protein digestion and LC-ESI-MS/MS analysis.......S8
8. **Computational Studies**.......S9
   8.1 Building of the *apo*-BAAG model.......S9
   8.2 Molecular Dynamics simulations of the *apo*-BAAG protein.......S11
   8.3 Docking studies.......S12
   8.4 Molecular Dynamics simulations of the BAAG/ligand complexes.......S12
   8.5 MM/PBSA calculations.......S12
   8.6 Water-swap calculations.......S13
9. **References**.......S13

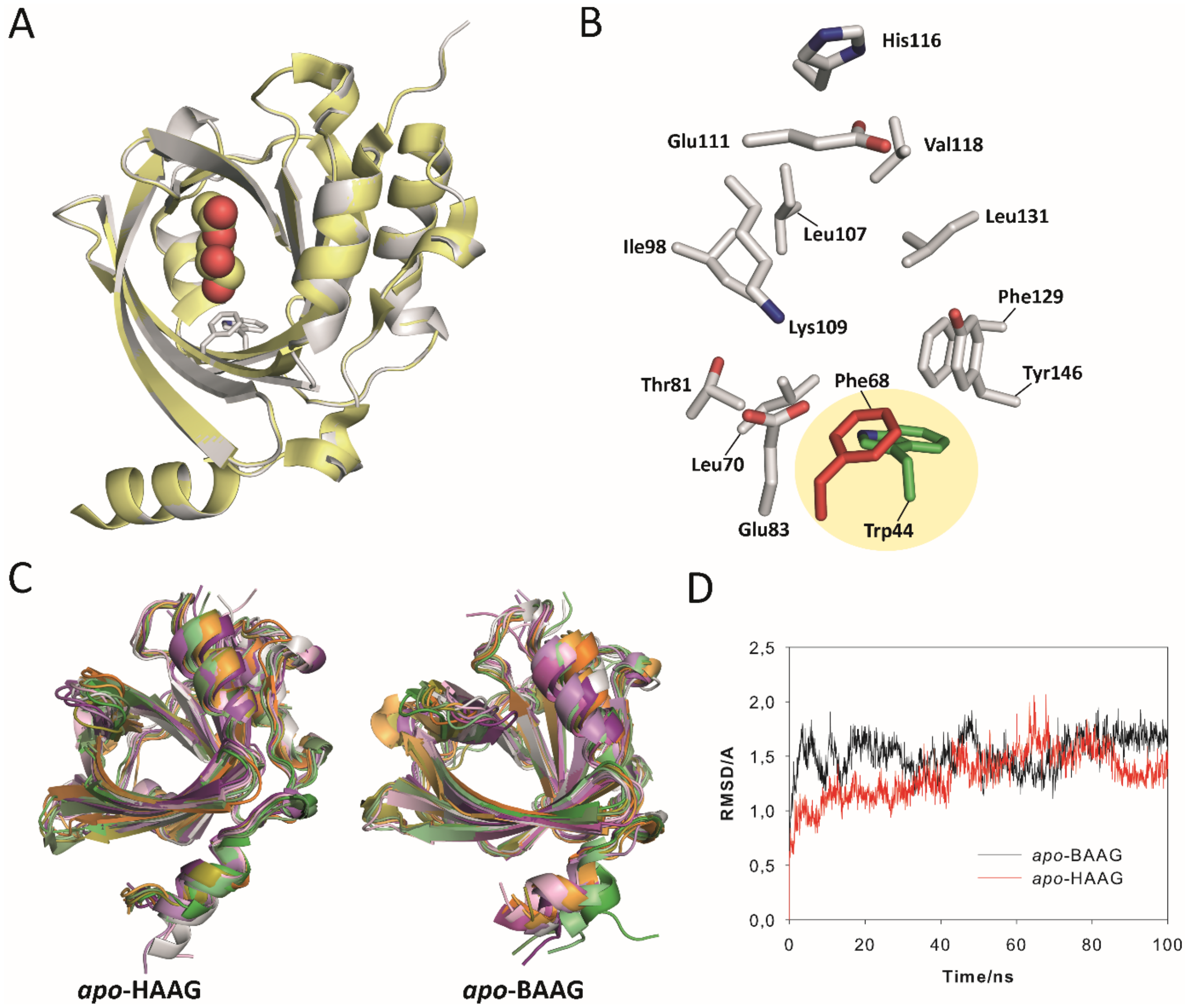

Figure S1. (A) Comparison of the crystal structure of the HAAG/(*R*)-1-glycerol acetate complex (PDB code 3KQ0, 1.8 Å, yellow) and the BAAG homology model (gray). (*R*)-1-glycerol acetate (yellow, spheres) in HAAG and Phe68 and Trp44 residues in BAAG are highlighted. (B) Detail view of the recognition center of the BAAG model. Relevant side chain residues are shown and labelled. Key residues Phe68 and Trp44 are highlighted in red and green, respectively. (C) Comparison of the several snapshots of the *apo*-HAAG and *apo*-BAAG proteins during 100 ns of MD simulations. Note how the tridimensional structure of the *apo*-BAAG protein is stable as the *apo*-HAAG one during the simulation. (D) RMSD plots for the protein backbone (Cα, C, N, and O atoms) in the *apo*-HAAG (red) and *apo*-BAAG (black) obtained from MD simulations studies.

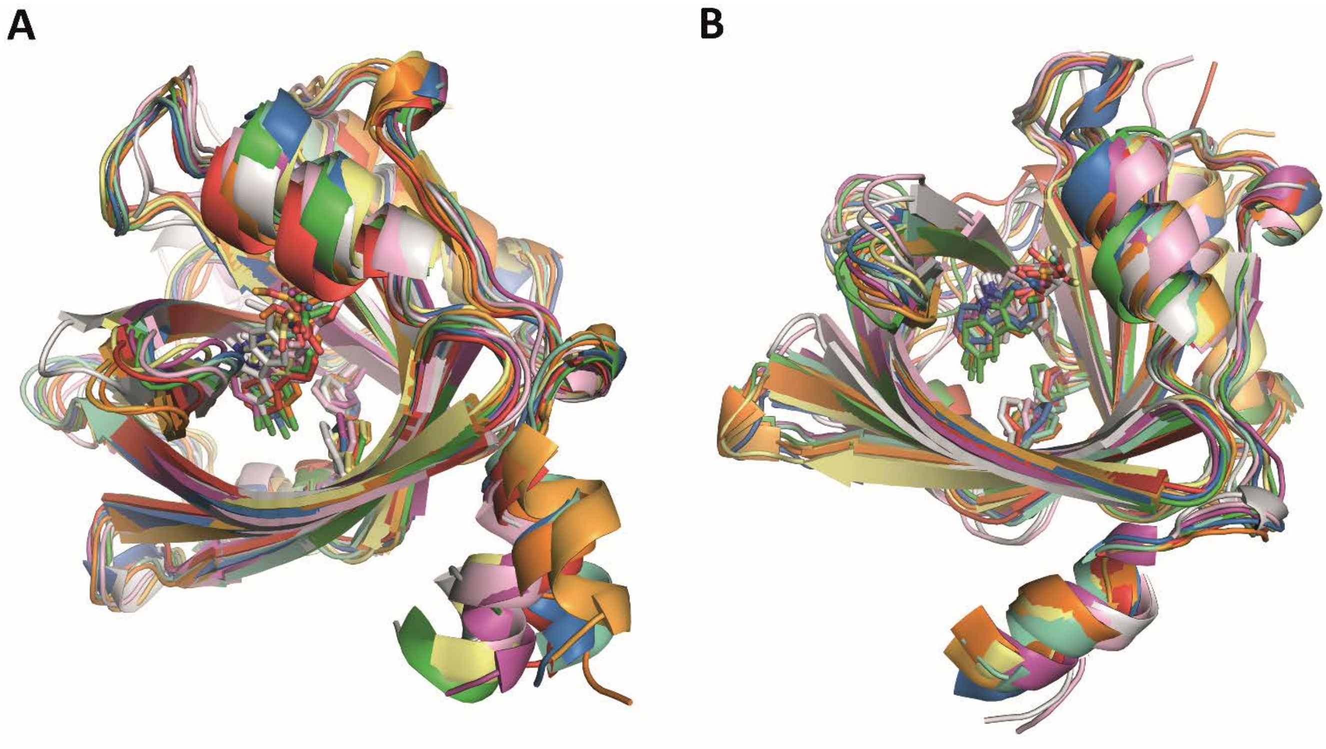

Figure S2. Comparison of several snapshots of the BAAG/(*S*)-CPFMe (A) and BAAG/(*R*)-CPFMe (B) protein complexes during 100 ns of MD simulations. The ligand and side chain residues of Phe68 and Phe129 are shown as sticks.

Table S1. Calculated Binding Free Energies using MM/PBSA

Ligand	Energy (kcal mol^{-1})	Relative Energy Difference (kcal mol^{-1})[a]
(*S*)-CPFMe	-29.9 ± 0.2[b]	0
(*R*)-CPFMe	-29.0 ± 0.2[b]	+0.9

[a]relative to the ligand of highest affinity

[b]standard error of mean

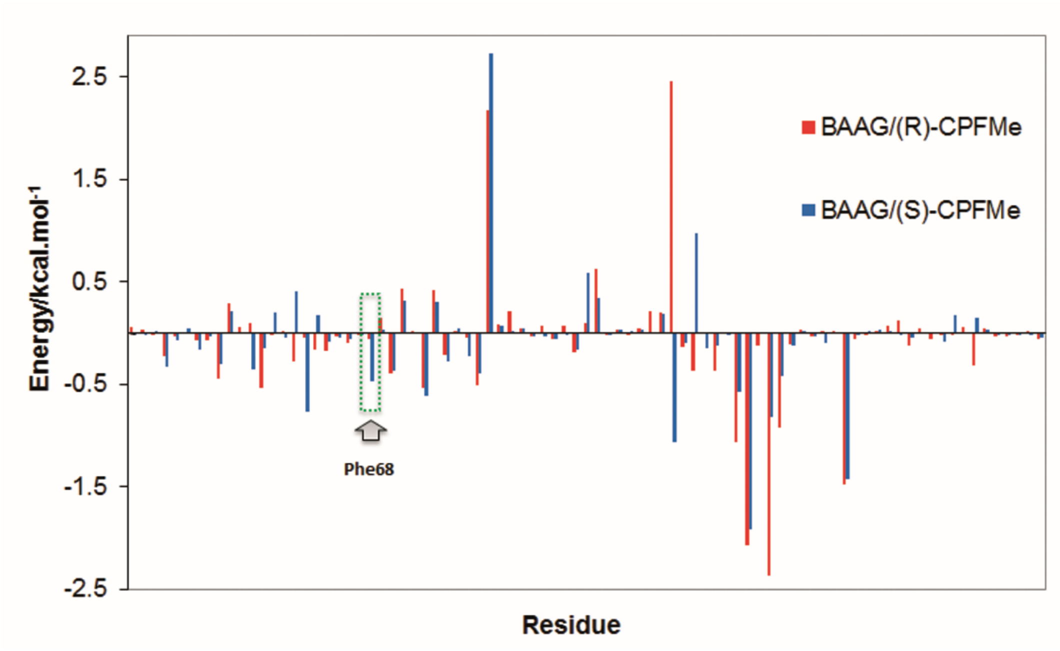

Figure S3. Plot of the calculated energy contribution per residue in the BAAG/(*S*)-CPFMe (red) and BAAG/(*R*)-CPFMe (blue) complexes obtained from MD simulations studies using water-swap. Note how the interaction of Phe68 residue with the *S* enantiomer is stronger than with its enantiomer.

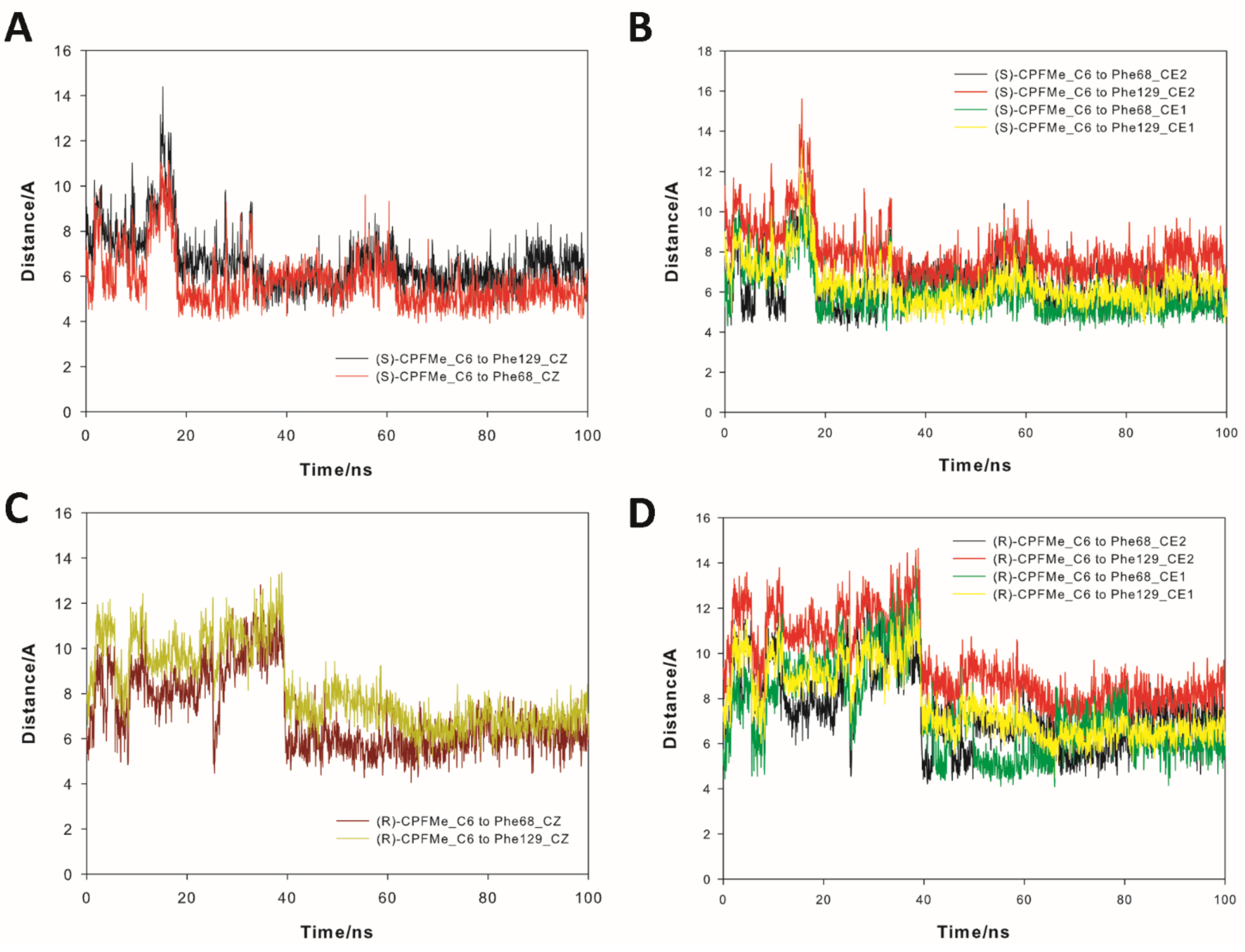

Figure S4. Comparison of the variation of the relative distance between the C6 atom of (*S*)-CPFMe (A,B) and (*R*)-CPFMe (C,D) and the CZ atom (*para*-) and the CE1 and CE2 atoms (*meta*-) of Phe68 and Phe129 in the corresponding BAAG/CPFMe protein complexes during whole simulation: (A) C6 atom of (*S*)-CPFMe *vs* CZ atoms of Phe68 and Phe129; (B) C6 atom of (*S*)-CPFMe *vs* CE1 and CE2 atoms of Phe68 and Phe129; (C) C6 atom of (*R*)-CPFMe *vs* CZ atoms of Phe68 and Phe129; (D) C6 atom of (*R*)-CPFMe *vs* CE1 and CE2 atoms of Phe68 and Phe129. Note how the C6 atom of the two enantiomers is located closer to Phe68 than Phe129 during the 100 ns of simulation. In addition, among the two isomers, (*S*)-CPFMe is located closer to the Phe68 than its enantiomer.

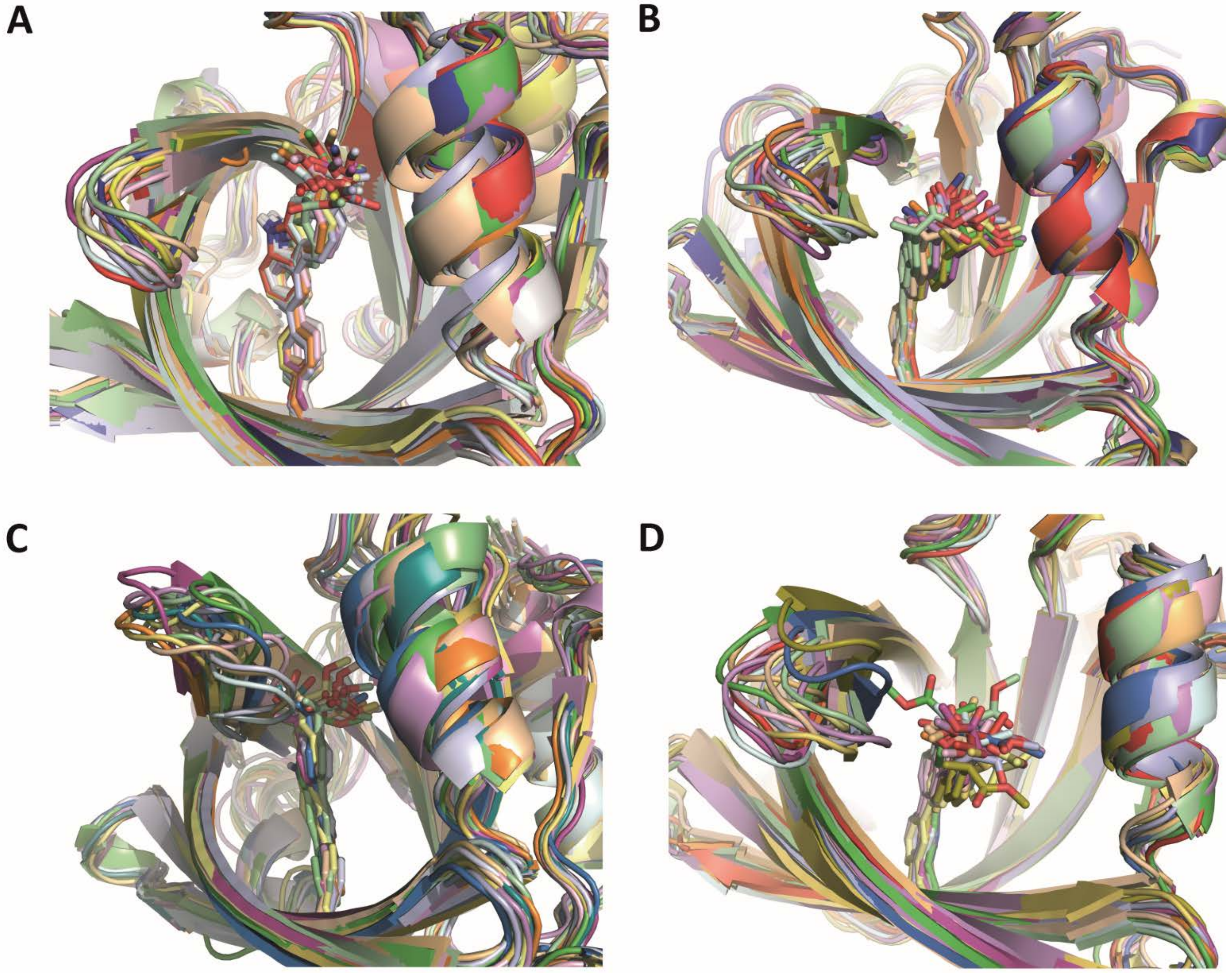

Figure S5. Comparison of the several snapshots of the BAAG/CPFMe adducts during 100 ns of MD simulations: (A,B) (*S*)-CPFMe (A,B) and (*R*)-CPFMe (C,D). The covalent modification or Phe68 at the *para*- (A,C) and *meta*- positions (B,D) of the phenyl ring are considered. The covalently modified Phe68 is shown as sticks.

7. Experimental Section

7.1 General – Racemic **CPF** and BAAG were commercially available. Spectrophotometric, HPLC or reagent grade solvents were used without further purification. Solutions of phosphate-buffered saline (PBS) (0.01 M, pH 7.4) were prepared by dissolving phosphate-buffered saline tablets in Milli-Q water. The ^{1}H-NMR and ^{13}C-NMR spectra were recorded in $CDCl_3$ at 400 and 100 MHz, respectively, using a Bruker AVANCE III instrument; chemical shifts (δ) are reported in ppm. Steady state absorption spectra were recorded in a JASCO V-630 spectrophotometer. Values are given for λ_{max} (in nm), with log ε (in brackets). Analytic HPLC analysis was performed by means of a Waters HPLC system connected to a PDA Waters 2996 detector. Optical rotation was measured in a JASCO P-1030 Polarimeter (c = 0.001 g/100 mL, CH_3CN), light source sodium (589 nm). Preparative HPLC isolation was carried out on a JASCO HPLC equipment, composed of a DG-2080-54 degasification system, LG-2080-04 mixer and a PU-2080 pump connected to a UV-1575 detector.

7.2 Synthesis of (*R*)-CPFMe and (*S*)-CPFMe – First, enantiopure (100%) (*R*)- and (*S*)-**CPF** were separated from a 1.8 M racemic mixture in methyl tert-butyl ether by HPLC (Technocroma Kromasil 100-TBB column, mobile phase hexane/methyl tert-butyl ether/acetic acid (45:55:0.1, v/v/v) flow 2.2 mL/min). Subsequently, to a suspension of enantiopure (*R*)- or (*S*)-**CPF** (50 mg, 0.18 mmol) in MeOH (5 mL), 5 drops of H_2SO_4 were added. The mixture was stirred overnight at room temperature. After evaporating the MeOH, the residue was taken in EtOAc and washed with $NaHCO_3$ (3 x 20 mL) and brine (10 mL). The organic phase was dried (anhydrous $MgSO_4$) and the solvent was evaporated to obtain quantitatively enantiopure (*R*)- or (*S*)-**CPFMe**, as white solids.

Data for (*R*)-**CPFMe**: $[\alpha]_D^{19}$ = -57º; UV: 239 (4.7), 299 (4.3), 330 (3.6) and 343 (3.5). ^{1}H NMR (δ, ppm): 8.07 (s, 1H), 8.00–7.97 (m, 1H), 7.95 (d, J = 8.1 Hz, 1H), 7.38–7.30 (m, 3H), 7.18 (dd, J_1 = 8.1, J_2 = 1.5 Hz, 1H), 3.91–3.86 (m, 1H), 3.68 (s, 3H), 1.59 (d, J = 7.2 Hz, 3H); ^{13}C NMR (δ, ppm): 175.3, 140.5, 139.5, 138.2, 126.0, 125.2, 124.5, 121.8, 120.8, 120.1, 119.8, 111.7, 109.7, 52.3, 46.0, 19.1; HRMS calcd for $C_{16}H_{13}NO_2{}^{35}Cl$: 286.0635 ($[M-H]^-$); found 286.0632.

Data for (*S*)-**CPFMe**: $[\alpha]_D^{19}$ = + 57º; UV: 239 (4.7), 299 (4.3), 330 (3.6) y 343 (3.5). ^{1}H NMR (δ, ppm): 8.10 (s, 1H), 7.99–7.97 (m, 1H), 7.94 (d, J = 8.1 Hz, 1H), 7.38–7.29 (m, 3H), 7.18 (dd, J_1 = 8.1, J_2 =1.5 Hz, 1H), 3.93–3.85 (m, 1H), 3.69 (s, 3H), 1.59 (d, J = 7.2 Hz, 3H); ^{13}C NMR (δ, ppm): 175.4, 140.5, 139.4, 138.2, 126.0, 125.1, 124.5, 121.8, 120.8, 120.1, 119.8, 111.7, 109.7, 52.3, 46.0, 19.1; HRMS calcd for $C_{16}H_{13}NO_2{}^{35}Cl$: 286.0635 ($[M-H]^-$); found 286.0639.

7.3 Fluorescence Experiments – Spectra were recorded on a JASCO FP-8500 spectrofluorometer system, provided with a monochromator in the wavelength range of 200-850 nm, at 22°C. Experiments were performed on solutions of (*R*)- or (*S*)-**CPFMe** (3.3 × 10-5 M) in the presence of BAAG (at 2:1 protein/**CPFMe** molar ratio), employing 10 × 10 mm^2 quartz cells with 4 mL capacity.

7.4 Laser flash photolysis experiments – A Q-switched Nd:YAG laser (Quantel Brilliant, 355 nm, 15 mJ per pulse, 5 ns fwhm) was coupled to a mLFP-111 Luzchem miniaturized equipment was employed. This transient absorption spectrometer includes a ceramic xenon light source, 125 mm monochromator, Tektronix 9-bit digitizer TDS-3000 series with 300 MHz bandwidth, compact photomultiplier, power supply, cell holder and fiber optic connectors, fiber optic sensor for laser-sensing pretrigger signal, computer interfaces, and a software package developed in the LabVIEW environment from National Instruments. The LFP equipment supplies 5 V trigger pulses with programmable frequency and delay. The rise time of the detector/digitizer is ~3 ns up to 300 MHz (2.5 GHz sampling). The monitoring beam is provided by a ceramic xenon lamp and delivered through fiber optic cables. The laser pulse is probed by a fiber that synchronizes the LFP system with the digitizer operating in the pretrigger mode. Transient spectra and kinetic traces were recorded employing 10 × 10 mm^2 quartz cells with 4 mL capacity. The concentration of CPFMe in was 3.3×10^{-5} M, and the CPFMe/protein molar ratio was 1:2. All the experiments were carried out at room temperature. The τT values of CPF were determined by fitting the decay traces at λ_{max} = 450 nm by means of a monoexponential function.

7.5 Steady-state Photolysis Experiments – Steady-state photolysis of (*S*)- or (*R*)-**CPFMe** (3.3×10^{-5} M) was performed by using a 150 W Xe lamp coupled to a monochromator at lamp output (λ_{exc} = 320 nm) in PBS under air and in the presence of protein (BAAG/**CPFMe**, 2:1 molar ratio), through Pyrex. was The course of the reaction was followed by monitoring the changes in the fluorescence spectra of the reaction mixtures at increasing times.

7.6 Treatment with guanidinium chloride and filtration through Sephadex – Guanidinium chloride (1.72 mL, 6 M) was added to 3 mL of (*R*)- or (*S*)-**CPFMe**@BAAG in PBS, in order to cause protein denaturation. The mixture was then filtered through a Sephadex P-10 column. Firstly, 25 mL of pure PBS were eluted; then 2.5 mL of the BAAG/**CPFMe** mixture treated with GndCl were eluted. Subsequently, 3.5 mL of PBS were eluted again. The absorption and emission of the final sample were then measured. To take into account the dilution factor, a similar experiment was conducted directly on BAAG (in the absence of **CPFMe**). In this way, the ratio between the absorbance value before and after filtration was obtained, which was employed as correction factor in the experiments.

7.7 Protein Digestion and LC-ESI-MS/MS Analysis –Bovine α1-acid glycoprotein was enzymatically digested into smaller peptides using trypsin. Subsequently, these peptides were analyzed using nanoscale liquid chromatography coupled to tandem mass spectrometry (nano LC-MS/MS). Briefly, 20 µg of sample were taken (according to Qubit quantitation) and the volume was set to 20 µL. Digestion was achieved with sequencing grade trypsin (Promega) according to the following steps: i) 2 mM DTT in 50 mM NH_4HCO_3 V = 25 µL, 20 min (60 °C); ii) 5.5 mM IAM in 50 mM NH_4HCO_3 V=30 µL, 30 min (dark); iii) 10 mM DTT in 50 mM

$NH4HCO3$ V = 60 µL, 30 min; iv) Trypsin (Trypsin: Protein ratio 1:20 w/w) V=64 µL, overnight 37 °C. Digestion was stopped with 7 µL 10 % TFA (Cf protein ca 0.28 µg/µL).

Next, 5 µL of sample (except the main bands) were loaded onto a trap column (NanoLC Column, 3µ C18–CL, 350 um x 0.5 mm; Eksigent) and desalted with 0.1% TFA at 3 µL/min during 5 min. The peptides were then loaded onto an analytical column (LC Column, 3 µ C18–CL, 75 um × 12 cm, Nikkyo) equilibrated in 5% acetonitrile 0.1% formic acid. Elution was carried out with a linear gradient of 5 to 45% B in A for 30 min (A: 0.1% formic acid; B: acetonitrile, 0.1% formic acid) at a flow rate of 300 µL/min. Peptides were analyzed in a mass spectrometer nanoESI qQTOF (5600 TripleTOF, ABSCIEX). The tripleTOF was operated in information-dependent acquisition mode, in which a 0.25-s TOF MS scan from 350–1250 m/z was performed, followed by 0.05-s product ion scans from 100-1500 m/z on the 50 most intense 2-5 charged ions.

ProteinPilot v4.5. (ABSciex) search engine default parameters were used to generate peak list directly from 5600 TripleTOF wiff files. The obtained mgf was used for identification with MASCOT (v 4.0, Matrix- Science). Database search was performed on SwissProt database. Searches were done with tryptic specificity allowing one missed cleavage and a tolerance on the mass measurement of 100 ppm in MS mode and 0.6 Da in MS/MS mode. Carbamidomethylation of Cys was used as a fixed modification and oxidation of Met and deamidation of Asn and Gln as variable modifications. A modification was defined in Phe, Tyr, Thr for **CPFMe**.

8. Computational Studies

8.1 Building of the *apo*-BAAG Model – The Phyre2 homology modelling web server was used to model the three-dimensional structure of BAGG.[16] The coordinates of the crystallographically determined human α1-acid glycoprotein (HAAG) (PDB code 3KQ0, 1.8 Å)[17] was chosen as the main template for building the model. The resulting BAAG structure had a 52.46% sequence identity and 85% of its sequence was modelled with 100% confidence by the template (Figures S1A and S6). A similar model was obtained by using the SWISS-MODEL Homology server (Figure S7).[20]

In order to evaluate the veracity of the constructed *apo*-BAAG model, MD simulation studies were performed. These studies were also carried out with the *apo*-HAAG as a control by using the protein coordinates of the crystal structure PDB code 3KQ0. The monomer of each protein immersed in a truncated octahedron of water molecules obtained using the molecular mechanics force field AMBER[21] was used. The results from 100 ns of dynamic simulation showed a larger motion in the region involving residues 83–116 of the *apo*-BAAG model than in the corresponding *apo*-HAAG protein. Reasoning that this fact would be due to some differences in the relative arrangement of some polar residues located in this region of the model that close the recognition centre, the terminal groups of residues Glu83, Gln85 and Asn94 were rotated 90º relative to the original arrangement. The latter was based on the relative disposition of these residues observed in the crystal structure of HAAG/chlorpromazine complex (PDB code 3APX,[22] 2.2 Å). In addition, the three possible protonation states of His116 were evaluated, i.e. single (ε or δ) and dual (ε and δ).

The best results, i.e. less motion, were achieved when the His116 is protonated at δ position. The resulting new *apo*-BAAG model proved to be more stable than the initial one showing a stability similar to the *apo*-HAAG protein (Figure S1D). This can also be clearly visualized by analysing the root-mean-square deviation (rmsd) of the protein backbone (Cα, C, N, and O atoms) in the two proteins, which in both cases proved to be low (Figure S1D).

```
BAAG_model:        MALLWALAVLSHLPLLDAQSPECANLMTVAPITNATMDLLSGKWFYIGSAFRNPEYKNSA
HAAG_3APX:         -----------------MQIPLCANLVP-VPITNATLDRITGKWFYIASAFRNEEYNKSV
HAAG_3KQ0:         ------------------EIPLCANLVP-VPITNATLDQITGKWFYIASAFRNEEYNKSV
                                       * ****    ****** *   ****** ***** **  *

BAAG_model:        RAIQAAFFYLEPRHAEDKLITREYQTIEDKCVYNCSFIKIYRQNGTLSKVESDREHFVDL
HAAG_3APX:         QEIQATFFYFTPNKTEDTIFLREYQTRQNQCFYNSSYLNVQRENGTVSRYEGGREHVAHL
HAAG_3KQ0:         QEIQATFFYFTPNKTEDTIFLREYQTRQDQCIYNTTYLNVQRENGTISRYVGGQEHFAHL
                     *** ***  *   **    *****    * **       * *** *      **   *

BAAG_model:        LLSKHFRTFMLAASWNGTKNVGVSFYADKPEVTQEQKKEFLDVIKCIGIQESEIIYTDEK
HAAG_3APX:         LFLRDTKTLMFGSYLDDEKNWGLSFYADKPETTKEQLGE---------------------
HAAG_3KQ0:         LILRDTKTYMLAFDVNDEKNWGLSVYADKPETTKEQLGE---------------------
                   *      * *        ** * * ****** * **  *

BAAG_model:        KDACGPLEKQHEEERKKETEASYEALDCLRIPRSDVMYTDWKKDKCEPLEKQHEKERKQE
HAAG_3APX:         ---------------------FYEALDCLRIPRSDVMYTDWKKDKCEPLEKQHEKERKQE
HAAG_3KQ0:         ---------------------FYEALDCLRIPKSDVVYTDWKKDKCEPLEKQHEKERKQE
                                         ********** *** ***********************

BAAG_model:        -------------
HAAG_3APX:         EGESHHHHHH---
HAAG_3KQ0:         EGESAWSHPQFEK
```

Figure S6. Amino acid sequence alignments for the BAAG homology model and two crystal structures of HAAG [PDB codes: 3APX (2.2 Å) and 3KQ0 (1.8 Å)]. Protein sequences were aligned using the CLUSTAL Omega multiple sequence alignment (**http://www.ebi.ac.uk/Tools/msa/clustalo/, accessed September 9, 2016**). Identical residues in the proteins used in these studies are highlighted in blue.

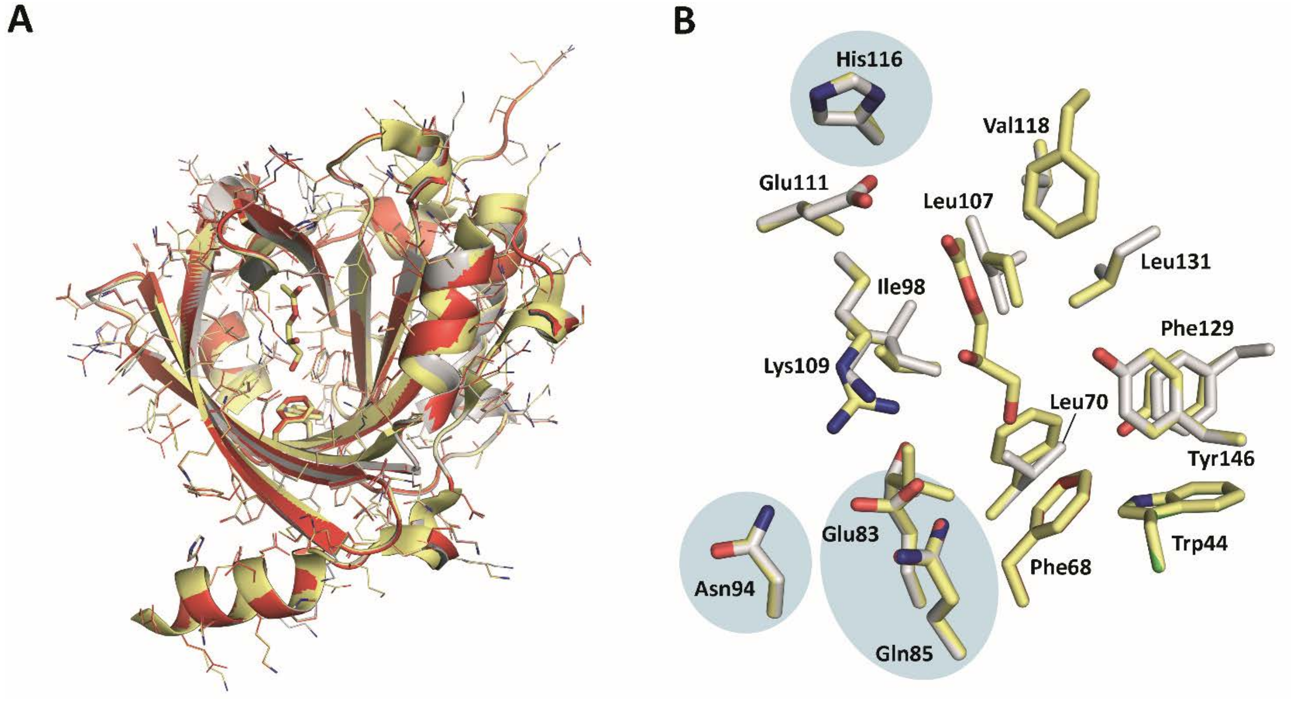

Figure S7. (A) Comparison of the crystal structure of the HAAG/(*R*)-1-glycerol acetate complex (PDB code 3KQ0, 1.8 Å, yellow) and the two BAAG homology models [Phyre´s model (gray) and Swiss´s model (red)]. (*R*)-1-glycerol acetate (yellow, sticks) in HAAG and Phe68 and Trp44 residues in BAAG are highlighted. (B) Comparison of the recognition center of the HAAG/(*R*)-1-glycerol acetate complex (yellow) and BAAG Phyre´s model (gray). The relevant side chain resides are shown. The labelling corresponds to the BAAG protein. The residues which disposition was modified from the initial homology model are highlighted with a blue circle.

8.2 Molecular Dynamics Simulations of the *apo*-BAAG Protein

(a) Minimization. Simulations of *apo*-BAAG form were carried out using the protein geometries obtained by homology modelling. Computation of the protonation state of titratable groups at pH 7.0 was carried out using the H++ Web server.[23] Addition of hydrogen and molecular mechanics parameters from the ff14SB[24] and GAFF force fields, respectively, were assigned to the protein and the ligands using the LEaP module of AMBER Tools 14.[25-26,21] As a result of these analysis, two possible protonation states of His99 were studied, i.e. in ε and δ position. In addition, the terminal groups of residues Glu66, Asn77 and Gln68 were rotated 90°. The protein was immersed in a truncated octahedron of ~10000 TIP3P water molecules and neutralized by addition of sodium ions.[25,27-28] The system was minimized in three stages: (a) minimization of the solvent and ions (5000 steps, first half using steepest descent and the rest using conjugate gradient); (b) minimization of the side chains, waters and ions (5000 steps, first half using steepest descent and the rest using conjugate gradient); (c) final minimization of the whole system (5000 steps, first half using steepest descent and the rest using conjugate gradient). A positional restraint force of 50 kcal mol^{-1} $Å^{-2}$ was applied to the whole protein and α carbons during the first two stages (a–b), respectively.

(b) Simulations. MD simulations were performed using the pmemd.cuda_SPFP[29-31] module from the AMBER 14 suite of programs. Periodic boundary conditions were applied and electrostatic interactions were treated using the smooth particle mesh Ewald method (PME)[32] with a grid spacing of 1 Å. The cutoff distance for the non-bonded interactions was 9 Å. The SHAKE algorithm[33] was applied to all bonds containing hydrogen, using a tolerance of 10–5 Å and an integration step of 2.0 fs. The minimized system was then heated at 300 K at 1 atm by increasing the temperature from 0 K to 300 K over 100 ps and by keeping the system at 300 K another 100 ps. A positional restraint force of 50 kcal mol^{-1} $Å^{-2}$ was applied to all α carbons during the heating stage. Finally, an equilibration of the system at constant volume (200 ps with positional restraints of 5 kcal mol^{-1} $Å^{-2}$ to α alpha carbons) and constant pressure (another 100 ps with positional restraints of 5 kcal mol^{-1} $Å^{-2}$ to α alpha carbons) were performed. The positional restraints were gradually reduced from 5 to 1 mol^{-1} $Å^{-2}$ (5 steps, 100 ps each), and the resulting systems were allowed to equilibrate further (100 ps). Unrestrained MD simulations were carried out for 100 ns. System coordinates were collected every 10 ps for further analysis. Figures depicting structures were prepared using PYMOL.[34] The cpptraj module in AMBER 14 was used to analyze the trajectories and to calculate the root-mean-square deviations (RMSD) of the protein during the simulation.[35] Clustering analysis showed that the conformation of the homology model is highly populated in the *apo* protein trajectory. As a result of these analysis, the three possible protonation states of His116 were studied, i.e. single (ε or δ) and dual (ε and δ). The best results were obtained with His116 protonated at δ position. In addition, the terminal groups of residues Glu66, Asn77 and Gln68 were rotated 90°.

8.3 Docking Studies

They were carried out using program GOLD 5.2[15] and the protein geometries of the homology model. Ligand geometries were minimized using the AM1 Hamiltonian as implemented in the program Gaussian 09[36] and used as MOL2 files. Each ligand was docked in 25 independent genetic algorithm (GA) runs, and for each of these a maximum number of 100000 GA operations were performed on a single population of 50 individuals. Operator weights for crossover, mutation and migration in the entry box were used as default parameters (95, 95, and 10, respectively), as well as the hydrogen bonding (4.0 Å) and van der Waals (2.5 Å) parameters. The position of (*R*)-1-glycerol acetate present in the crystal structure of the human α1-acid glycoprotein (PDB 3KQ0) was used to define the active-site and the radius was set to 8 Å. The "flip ring corners" flag was switched on, while all the other flags were off. The GOLD scoring function was used to rank the ligands in order to fitness.

8.4 Molecular Dynamics Simulations Studies of the BAAG/ligand Complexes

(a) Ligand preparation. The ligand geometries of the highest score solution obtained by docking were minimized using a restricted Hartree–Fock (RHF) method and a 6–31G(d) basis set, as implemented in the ab initio program Gaussian 09. Ligands were manually docked into the active site as it was obtained by docking. The resulting wavefunctions were used to calculate electrostatic potential-derived (ESP) charges employing the restrained electrostatic potential (RESP)[37] methodology, as implemented in the assisted model building with energy refinement (AMBER)[21] suite of programs. The missing bonded and non-bonded parameters were assigned, by analogy or through interpolation, from those already present in the AMBER database (GAFF).[26]

(b) Minimization of binary complexes. The complex immersed in a truncated octahedron of TIP3P water molecules and sodium ions was minimized in four stages: (1) initial minimization of the ligand (500 steps, first half using steepest descent and the rest using conjugate gradient); steps (2), (3) and (4) were performed as steps (a), (b) and (c) in the *apo*-protein minimization (see above). A positional restraint force of 50 kcal mol^{-1} $Å^{-2}$ was applied to those unminimized atoms during the first three stages (1–3).

(c) Simulations. MD simulations of the binary complexes were performed as indicated for the *apo*-BAAG protein (see above).

8.5 MM/PBSA Calculations – The binding free energy for each ligand was calculated by the MM/PBSA[18] approach implemented in Amber Tools 1.5 ante-MMPBSA.py module was used to create topology files for the complex, receptor and ligand and binding free energies were calculated with the MMPBSA.py module.[38] A single trajectory approach was used to calculate binding free energies considering only the last 60 ns (302 snapshots) of the 100 ns MD trajectories (BAAG/ligand). The Poisson-Boltzmann (PB) and Generalized Born (GB) implicit solvation models were employed. The latter model provided relative free energy values more in agreement with the experimental results.

8.6 Water-swap Calculations – Binding free energies for both ligands were calculated using Water-swap application of Sire program (sire_14_4 version).[19] Cartesian coordinates obtained from the MD simulation of both ligands, from 20 ns to 100 ns every 10 ns (9 for each ligand), were used as a starting point in the binding free energy calculation. As Water-swap needs a cubic box of waters and MD simulations were run in a truncated octahedric box, around 11000 waters were added to every set of coordinates with tLeap module of AMBER 15. In all cases, this additional waters are far from the active site and do not interfere with Water-swap calculations. The average binding free energies and residue free energy components for both ligands were calculated with some outlier values discarded after careful examination.

9. References

15 http://www.ccdc.cam.ac.uk/solutions/csd-discovery/components/gold/

16 L. A. Kelley, S. Mezulis, C. M. Yates, M. N. Wass, and M. J. E. Sternberg, *Nature Protocols*, 2015, **10**, 845.

17 D. L. Schonfeld, R. B. Ravelli, U. Mueller and A. Skerra, *J. Mol. Biol.*, 2008, **384**, 393.

18 B. R. Miller III, T. D. McGee Jr., J. M. Swails, N. Homeyer, H. Gohlke and A. E. Roitberg, *J. Chem. Theory Comput.*, 2012, **8**, 3314.

19 (a) C. J. Woods, M. Malaisree, S. Hannongbua and A. J. Mulholland, *J. Chem. Phys.,* 2011, **134**, 054114. (b) C. J. Woods, M. Malaisree, J. Michel, B. Long, S. McIntosh-Smith and A. J. Mulholland, *Faraday Discussions*, 2014, **169**, 477.

20 (a) M. Biasini, S. Bienert, A. Waterhouse, K. Arnold, G. Studer, T. Schmidt, F. Kiefer, T. G. Cassarino, M. Bertoni, L. Bordoli and T. Schwede, *Nucleic Acids Res.*, 2014, **42** (W1), W252. (b) K. Arnold, L. Bordoli, J. Kopp and T. Schwede, *Bioinformatics*, 2006, **22**, 195. (c) P. Benkert, M. Biasini and T. Schwede, *Bioinformatics*, 2011, **27**, 343.

21 D. A. Case, J. T. Berryman, R. M. Betz, D. S. Cerutti, T. E. Cheatham, III, T. A. Darden, R. E. Duke, T. J. Giese, H. Gohlke, A. W. Goetz, N. Homeyer, S. Izadi, P. Janowski, J. Kaus, A. Kovalenko, T. S. Lee, S. LeGrand, P. Li, T. Luchko, R. Luo, B. Madej, K. M. Merz, G. Monard, P. Needham, H. Nguyen, H. T. Nguyen, I. Omelyan, A. Onufriev, D. R. Roe, A. Roitberg, R. Salomon-Ferrer, C. L. Simmerling, W. Smith, J. Swails, R. C. Walker, J. Wang, R. M. Wolf, X. Wu, D. M. York and P. A. Kollman (2015), AMBER 2015, AMBER 2015, Amber Tools 1.5; University of California, San Francisco.

22 The X-ray crystal structure is available from the Protein Data Bank (PBD: 3APX): K. Nishi,T. Ono, T. Nakamura, N. Fukunaga, M. Izumi, H. Watanabe, A. Suenaga, T. Maruyama, Y. Yamagata, S. Curry and M. Otagiri, Crystal structure of the A variant of human alpha1-acid glycoprotein and chlorpromazine complex (to be published).

23 (a) J. C. Gordon, J. B. Myers, T. Folta, V. Shoja, L. S. Heath and A. Onufriev, *Nucleic Acids Res.*, 2005, **33** (Web Server issue):W368. (b) http://biophysics.cs.vt.edu/H++.

24 J. A. Maier, C. Martinez, K. Kasavajhala, L. Wickstrom, K. E. Hauser and C. Simmerling, *J. Chem. Theory Comput.*, 2015, **11**, 3696.

25 D. A. Case, T. E. Cheatham, T. Darden, H. Gohlke, R. Luo, K. M. Merz, O. Onufriev, C. Simmerling, B. Wang and R. J. Woods, *J. Comput. Chem*., 2005, **26**, 1668.

26 (a) J. Wang, R. M. Wolf, J. W. Caldwell, P. A. Kollman and D. A. Case, *J. Comp. Chem*., 2004, **25**, 1157. (b) J. Wang, W. Wang, P. A. Kollman and D. A. Case*, J. Mol. Graphics Modell.*, 2006, **25**, 247.

27 J. Aqvist, *J. Phys. Chem*., 1990, **94**, 8021.

28 W. L. Jorgensen, J. Chandrasekhar and J. D. Madura, *J. Chem. Phys*., 1983, **79**, 926.

29 A. W. Goetz, M. J. Williamson, D. Xu, D. Poole, S. Le Grand and R. C. Walker, *J. Chem. Theory Comput.*, 2012, **8**, 1542.

30 R. Salomon-Ferrer, A. W. Goetz, D. Poole, S. Le Grand and R. C. Walker, *J. Chem. Theory Comput*., 2013, **9**, 3878.

31 S. Le Grand, A. W. Goetz, and R. C. Walker, *Comp. Phys. Comm.*, 2013, **184**, 374.

32 T. A. Darden, D. York and L. G. Pedersen, *J. Chem. Phys.*, 1993, **98**, 10089.

33 J.-P. Ryckaert, G. Ciccotti and H. J. C. Berendsen, *J. Comput. Phys.*, 1977, **23**, 327.

34 W. L. DeLano, The PyMOL Molecular Graphics System; DeLano Scientific LLC: Palo Alto, CA, 2008; http://www.pymol.org/

35 D. R. Roe and T. E. Cheatham, *J. Chem. Theory Comput*., 2013, **9**, 3084.

36 M. J. Frisch, G. W. Trucks, H. B. Schlegel, G. E. Scuseria, M. A. Robb, J. R. Cheeseman, G. Scalmani, V. Barone, B. Mennucci, G. A. Petersson, H. Nakatsuji, M. Caricato, X. Li, H. P. Hratchian, A. F. Izmaylov, J. Bloino, G. Zheng, J. L. Sonnenberg, M. Hada, M. Ehara, K. Toyota, R. Fukuda, J. Hasegawa, M. Ishida, T. Nakajima, Y. Honda, O. Kitao, H. Nakai, T. Vreven, J. A. Montgomery, Jr., J. E. Peralta, F. Ogliaro, M. Bearpark, J. J. Heyd, E. Brothers, K. N. Kudin, V. N. Staroverov, R. Kobayashi, J. Normand, K. Raghavachari, A. Rendell, J. C. Burant, S. S. Iyengar, J. Tomasi, M. Cossi, N. Rega, J. M. Millam, M. Klene, J. E. Knox, J. B. Cross, V. Bakken, C. Adamo, J. Jaramillo, R. Gomperts, R. E. Stratmann, O. Yazyev, A. J. Austin, R. Cammi, C. Pomelli, J. W. Ochterski, R. L. Martin, K. Morokuma, V. G. Zakrzewski, G. A. Voth, P. Salvador, J. J. Dannenberg, S. Dapprich, A. D. Daniels, Ö. Farkas, J. B. Foresman, J. V. Ortiz, J. Cioslowski, and D. J. Fox, Gaussian 09, Revision D.01, Gaussian, Inc.: Wallingford CT, 2009.

37 W. D. Cornell, P. Cieplak, C. I. Bayly, I. R. Gould, K. M. Merz, D. M. Ferguson, D. C. Spellmeyer, T. Fox, J. W. Caldwell and P. A. Kollman, *J. Am. Chem. Soc*., 1995, **117**, 5179.

38 http://www.amber.utah.edu/AMBER-workshop/London-2015/pca/
